# Supplementary material for: Are carbon emissions trading and green financial instruments synergistic? -Comprehensive quantitative research based on content analysis
Source: PLoS One. 2024 Mar 7;19(3):e0298601. doi: 10.1371/journal.pone.0298601 (PMC10919601; doi:10.1371/journal.pone.0298601)
Supplement: S1 Data — (ZIP) [file pone.0298601.s001.zip › Data/地级市Z_F工作报告.zip!/╦─┤¿/╦∞─■╩╨/└┤╫╘╣1⁄2╓┌║┼í╛┬φ┐╦╩2╛▌═°í┐_2018.docx]

各位代表：

我代表市人民政府，向大会报告工作，请予审议，并请市政协委员和其他列席会议的同志提出意见。

2017年工作回顾

2017年，在省委、省政府和市委的坚强领导下，在市人大、市政协的监督支持下，我们认真学习领会习近平新时代中国特色社会主义思想，深入落实省委“加快发展、弯道超车”新要求，大力推进富民强市“七大提升行动”，全市经济社会持续健康发展。全年实现地区生产总值1138.06亿元、同比增长8.3%，地方一般公共预算收入60.18亿元、同口径增长13.88%，城乡居民人均可支配收入分别增长8.7%和9.3%。特别是，我们接续奋斗，通过十多年不懈努力，成功创建全国文明城市，夺得全国社会治安综合治理“长安杯”。

一年来，我们着力稳定增长，在聚力蓄势中实现新突破

投资拉动力持续增强。落实“项目年”部署，完成全社会固定资产投资1257.6亿元、增长11.7%。狠抓120个省、市重点项目建设，喜之郎、广义微电子等项目实现竣工，宋瓷文化中心、田家渡大桥等项目顺利开工，创维西部产业园等项目加快推进。集中开工四批次项目643个、估算总投资1765亿元。争取中省预算内投资项目101个、补助资金12亿元。累计签约PPP项目13个，吸引社会资本投资114亿元。

消费牵引力持续提升。积极参加省级市场拓展“三大活动”，社会消费品零售总额增长12%。成功举办第十届观音文化旅游节、第31届全国荷花展等41个节会活动，实现旅游总收入385亿元、增长24.4%。顺应消费升级趋势，推进“放心舒心消费城市”建设，在全省率先建立“绿色遂宁·放心舒心消费”信息化平台，已入驻商家420户。

开放带动力持续上升。实施“精准招商年”活动，到位市外资金479亿元。积极参加西博会进出口展等展会活动，举办或承办第十六届海科会主题市推介会、第八届泛成渝经济区商会合作峰会暨首届遂商大会、全国物流园区工作年会、中国仓储与配送协会企业家年会等活动，引进天翔环境等投资10亿元以上项目18个。与成都市、重庆市潼南区签署协议，在基础设施共建等方面开展务实合作。引入民营企业共建欧美产业园。全面启动通关一体化，海关监管货物量增长86.3%。

一年来，我们着力做大产业，在调转结合中取得新进展

产业结构不断优化。三次产业结构调整为14.1:48.2:37.7。规上工业增加值增长9.7%，“4 2”产业总产值增长19.8%，沱牌舍得利润总额实现翻番，江淮汽车下线整车2.5万辆，磨溪天然气净化厂处理量达到90亿方，新增规上企业45户，船山区和大英县入选全省第二批工业强县示范县（区）。服务业增加值增长10%，中国西部现代物流港营业收入突破500亿元，仁里古镇等3个项目入围全国优选旅游名录。新建高标准农田24.4万亩，粮食总产稳步增长。“遂宁鲜”通过国家工商总局商标注册审核，并获评全省十大优秀农产品区域公用品牌。

产业创新不断强化。高新技术产业总产值突破500亿元。国家知识产权试点城市通过验收，国家创新型城市和省级高新技术产业园区创建通过专家评审，遂宁经开区电子电路产业集群被纳入国家创新型产业集群试点。四川石墨烯应用产业技术研究院、中国生态酿酒产业技术研究院等挂牌成立，赛伯乐（遂宁）双创中心正式开园。在全省率先建成知识产权举报投诉与维权援助中心。之江高新荣获第十九届中国专利优秀奖，高金公司荣获四川质量奖提名奖。新增国家级高技能人才培训基地1个、国家高新技术企业20家、国家知识产权优势企业7家。

产业融合不断深化。鼓励“互联网 ”新业态发展，在全省率先颁发网约车平台许可证，累计投放共享单车1.2万辆，实现电商交易额61.8亿元、增长40.5%。落实“宽带中国”战略，完成国家第二批电信普遍服务试点，新增和升级395个通光纤行政村。推动工业化信息化深度融合，飞亚动力、沱牌舍得入围全国“两化融合”贯标试点。深化军地共建和军民融合，“遂宁舰”正式入列，8家企业取得军工标认证。

一年来，我们着力做美城乡，在统筹兼顾中展现新风貌

枢纽地位进一步凸显。交通基础设施建设完成投资36.5亿元。涪江五桥、城市中环线东南半环、遂宁至大英快捷通道等项目相继建成。西部铁路物流园铁路专用线与国家铁路成功并轨。遂宁机场迁建项目主体工程开工建设。遂德高速、成遂南达万高铁、绵遂内城际铁路等重点项目前期工作稳步推进。

城镇功能进一步完善。城镇提升行动项目完成投资118.8亿元，预计常住人口城镇化率提高1.3个百分点。国家海绵城市试点综合考评蝉联全国第2名，成功承办全国试点工作现场会。“多规合一”有序落实，中心城区控制性详细规划实现全覆盖。五彩缤纷北路景观带、席吴二洲湿地公园提升改造等重点项目开工建设。15个生态小镇和5个花园小镇建设全面启动，安居区拦江镇入选全国特色小镇。

农村面貌进一步改善。新改建通村公路136.9公里，实施农村公路改善工程137.4公里，173公里农业大环线已有145公里具备通车条件。在全省率先启动“农村土坯房三年整治行动”，累计投入资金5.8亿元、完成整治22285户，经验在全省推广。扎实推动幸福美丽新村和“四好村”建设，全市幸福美丽新村达823个，入选省级“四好村”102个，创建第五届全国文明村3个。安居区创建成为全省“四好农村路”示范县，蓬溪县拱市村入围全国环境整治示范村和首批全国农村幸福社区建设示范单位。

一年来，我们着力补齐短板，在攻坚克难中满足新需要

深化改革补齐供给短板。完成重点改革任务76项，推进中省层面改革试点36项。减税降费力度持续加大，全市共减免各项税收20.2亿元，营改增后综合税负由3.45%降为2.89%。引导银行机构发布“增投入、降成本、稳增长”十条倡议，推动设立首期10亿元产业发展基金、3亿元遂商返乡发展基金，为企业提供应急转贷资金22.5亿元。制定促进房地产业健康发展十四条措施，

商品住房库存下降22.3%。扎实推进林业、城管、乡镇综合执法改革。深入推进行政审批和政务服务标准化、规范化建设，在船山、射洪开展相对集中行政许可权改革试点，全面推行“多证合一”试点，企业和个体工商户总数分别增长25.1%、23.5%。

精准脱贫补齐小康短板。脱贫攻坚再战再胜，高质量完成2.5万人脱贫、90个贫困村退出，贫困发生率由2016年的2.6%降至1.8%。“四项扶贫基金”达2.3亿元、增长26.6%。成功承办全省农业产业扶贫现场会，“村村入园、户户入社”和“产联式合作”经验被《人民日报》等媒体深度报道。建成易地扶贫搬迁住房4023套，实施搬迁11734人。选派“金融村官”293名，组织100名优秀教师轮岗支教。贫困人口医保“一个100%、两个10%控费”全面落实。社会扶贫成效明显，我市成为中国社会扶贫网首批试点市。

保护环境补齐生态短板。积极配合中央和省级环保督察，及时整改环保问题，建立区域污染联防联控机制，环境质量改善明显。综合施策治理大气污染，开展秸秆禁烧和综合利用专项行动，淘汰燃煤锅炉281.8蒸吨，全年空气质量优良天数达301天，PM2.5、PM10年均浓度同比分别下降12%、5%。启动城镇污水和城乡垃圾处理设施建设三年推进行动，遂宁市城市生活垃圾环保发电厂投入运行。关停或搬迁禁养区养殖场（户）758家，完成限适养区治理916家。扎实推进大规模绿化全川遂宁行动，完成营造林18.4万亩。创新设立“河长 警长”治水模式，涪江出川断面水质达标率达100%。

一年来，我们着力下沉服务，在惠民利民中创造新成绩

民生实事全面推进。投入民生资金159.3亿元，占一般公共预算支出的69.1%。全面完成九项民生工程、16件民生实事。扎实推进“百姓安居工程”，开工改造棚户区17523套。完成农村饮水安全工程投资4966.8万元，受益人口达19.5万人。集中整治“群众最不满意的10件事”，累计投入整治资金3.9亿元，相关事项有效投诉下降79%。

社会事业全面进步。新改扩建公办幼儿园19所，改扩建城市义务教育学校12所，改善城乡校舍8.7万平方米，“三免一补”惠及24.1万名学生，教育公共服务体系更加完善。全面取消公立医院药品加成，启动免费健康体检试点，成立3所市直医院乡镇分院，在全国首创建设联村卫生室，有效应对H7N9疫情。承办第二届西部优秀曲艺节目展演，举办市第六届运动会等活动，文化体育事业蓬勃发展。

社会保障全面加强。城镇新增就业3.9万人，城镇登记失业率控制在4.02%以内。完成全民参保登记，实现城乡居民基本医疗保险市级统筹，妥善解决近8万名被征地农民养老保险遗留问题。城乡居民低保标准分别提高到460元/月、275元/月。建立残疾学生助学金制度，发放残疾人“三项补贴”5864万元。新增养老床位2890张，建设城乡社区日间照料中心152个，我市被列为全国居家和社区养老服务改革试点地区。

一年来，我们着力促进和谐，在依法治理中获得新提升

依法行政深入实施。自觉接受市人大法律监督、工作监督，严格落实重大事项向人大及其常委会报告制度。主动接受市政协民主监督，积极向市政协通报情况。人大代表建议、政协委员提案办理满意和基本满意率达100%。起草完成的我市首部实体性地方法规《遂宁市城市管理条例》经市人大常委会审议通过，并报省人大常委会审批。成立遂宁市法律顾问团，各县（区）、市直各部门全面建立法律顾问制度，受理行政复议案件66件。加强审计工作，严格规范权力行使。

社会治理深入推进。全市刑事案件警情数、案件数分别下降26.2%和20.6%，破获数上升46.7%。加强农村技防建设，累计建成农村“雪亮工程”952处。市食品药品检验检测中心建成投用，基层食药监管所标准化建设率达91%，蓬溪县、大英县成功创建首批四川省食品安全示范县。新创建省级安全社区5个，安全生产主要控制指标持续下降，较大生产安全事故“零发生”。建成“12348”法律服务热线，办理法律援助案件1907件，涉法涉诉信访总量下降16.8%。“12345”政府服务热线居全国“百分百接通榜”第1　位、“城市总体排行榜”第2位。

作风建设深入开展。严格落实中央八项规定精神和省、市十项规定，“三公”经费同比下降9.2%。制定《精文简会高效办事十条措施》，政府发文数量下降19.9%。推进招投标、扶贫等领域突出问题专项整治，出台政府投资项目招投标监管六条措施，节资率提高5.87个百分点。强化行政监察，推动“阳光问廉”常态化。

统筹做好了国防动员、人防、民兵预备役、民族宗教、气象、保密、档案、机关事务管理、外事侨务、地方志、妇女儿童和关心下一代等工作。

各位代表，过去一年的成绩来之不易。这是省委、省政府和市委坚强领导的结果，是市人大、市政协监督支持的结果，是全市广大干部群众共同努力的结果。在此，我代表市人民政府，向全市人民，向人大代表、政协委员、各民主党派、工商联、无党派人士和各人民团体，向离退休老同志和所有关心支持遂宁发展的各界朋友，表示最衷心的感谢并致以最崇高的敬意！

各位代表，我们也清醒认识到，当前我市经济社会发展还存在一些矛盾和问题。经济发展不平衡不充分，提升质量、做大总量的“双重任务”没有改变，服务业和战略性新兴产业占比不足，新引进的大项目、好项目较少，部分企业生产经营困难，创新驱动力仍然不强；城乡发展不平衡不充分，城乡居民收入差距仍然较大，脱贫攻坚任务艰巨，教育、医疗、文化、养老等公共服务体系有待进一步完善；不稳定风险依然存在；一些政府部门和工作人员担当意识、服务意识不强，不作为、乱作为等现象还不同程度存在。对此，我们一定直面问题，以事不避难的决心、担当进取的作风，奋力开创政府工作新局面！

2018年主要任务

2018年是全面贯彻落实党的十九大精神的开局之年，是改革开放40周年，是大力实施“十三五”规划、决战决胜全面小康、建设绿色经济强市、实现美丽繁荣和谐遂宁目标承上启下的关键一年。今年政府工作的总体要求是：以习近平新时代中国特色社会主义思想为指导，深入贯彻党的十九大和省第十一次党代会、省委十一届二次全会精神，全面落实市委七届五次全会部署，牢牢把握稳中求进工作总基调，认真践行新发展理念，按照高质量发展要求，以供给侧结构性改革为主线，大力实施“四大发展战略”，纵深推进“七大提升行动”，坚决打好“三大攻坚战”，加快建设现代化经济体系，确保全市经济社会持续健康发展。

今年全市经济社会发展主要预期目标：地区生产总值同比增长8.5%，全社会固定资产投资增长10%，社会消费品零售总额增长12%，地方一般公共预算收入同口径增长9%，城乡居民人均可支配收入分别增长8.3%和9.3%，环境保护和节能减排降碳完成省政府下达任务。重点抓好以下工作。

一、狠抓转型升级，增强产业发展实力

推动制造业提质增效。力争“4 2”产业实现总产值1100亿元，规上工业增加值增长9.8%。推动园区升级，开展园区“竞进拉练”活动，做大做强国家级遂宁经济技术开发区，支持射洪、大英、安居、蓬溪4个重点工业园区创建省级开发区。狠抓工业项目，强化项目建设联席调度，确保绿然科技锂电池、广瑞半导体等80个市级重点工业项目顺利推进。夯实龙头支撑，实施“45520”大企业培育计划，引导盛马化工、美丰集团、川中建材等加快战略重组，支持天齐锂业、沱牌舍得、江淮汽车等龙头企业释放产能，集聚关联企业共同发展。做强中小企业，创建省级“专精特新”“高成长型”和“小巨人”企业25户，新增规上企业40户以上。

推动服务业提速扩量。力争服务业增加值增长10.3%。积极融入四川自贸区和重庆自贸区，加快“单一窗口”建设，提高通关便利化水平。深入推进全域旅游，实施“旅游 ”战略，加快浪漫地中海、沐水小镇等20个重点旅游项目建设，推动观音故里旅游区创建国家5A级景区。加大旅游营销力度，推广四大精品旅游线路，加快打造“成渝周末休闲首选地”。发展家政服务、社区服务，培育个性化、定制化、精细化服务。启动“消费升级行动计划”，积极参与省级市场拓展“三大活动”，全域深化“放心舒心消费城市”试点工作，完成“十百千”示范创建工程。

推动新经济融合壮大。出台促进新经济发展实施方案。积极支持新能源汽车相关产业发展，科学制定充电设施专项规划。启动工业互联网三年行动计划，新增“两化融合”贯标示范试点企业3户。推动二三产业共融发展，鼓励支持制造企业将运输、仓储等业务剥离外包。深化“互联网 商贸”发展，推动实体零售创新转型，打造一刻钟便民服务圈。推进电商和快递进农村，深化“农商互联”“电商快递互补”，畅通工业品下乡、农产品进城，力争电商交易额增长25%以上。推动军民融合深度发展，建立军民融合产业孵化基地，力争军工标认证企业达到10家。

二、狠抓改革创新，增添实体经济动力

打好科技创新攻坚战。全面推进国家创新型城市、国家知识产权示范城市、国家高新技术产业开发区创建工作。强化创新体系建设，支持新型研发机构发展，争取重大科技基础设施和国家实验室在遂布局。优化创新环境，用好“遂州科创贷”等科技金融产品，创新知识产权保护机制和开发利用模式，开展知识产权质押融资试点，新增国家高新技术企业10家以上。深化“创业遂州”行动，引进国科创新、启迪控股等孵化平台，支持建设创客公园、创业大街。大力“招才引智”，优化遂州英才“七大计划”，推进“周末名师”等柔性引才，力争新引进本科以上学历专业人才3500人以上。建设质量强市，培育60家企业争创第十三届四川名牌。尊重和激发企业家精神，弘扬劳模精神和工匠精神，加快新时代产业工人队伍建设，培训职业技能人才5000人。

深化重点领域改革。全面完成中省部署的改革试点任务，积极探索一批“遂宁经验”。深化供给侧结构性改革，修订出台降成本、促转型政策措施，全面落实减税降费政策，发展多层次资本市场，用好首期10亿元的产业发展基金等引导基金，进一步降低物流、融资、用能等成本，切实减轻企业负担。下大力气处置“僵尸企业”，加快盘活闲置资源。建立完善现代企业制度，激发国资国企和民营企业活力。推进财税、投融资和金融体制改革，调整完善分税制财政体制，鼓励金融机构争取投贷联动试点。深化工业用地弹性供应改革，推动低效工业用地处置及工矿废弃地复垦利用，实施农村建设用地整理及调整村庄建设用地布局试点。加强统计工作。

加力“放管服”改革。“放”出活力，深化行政审批制度改革，再取消下放一批行政审批事项。放宽市场准入，深化“多证合一、一照一码”，落实负面清单事项。“管”出公平，加快综合行政执法改革，用好投资项目在线审批监管平台，推行“双随机、一公开”监管，试点开展企业实名登记制度改革，严厉整治制售假冒伪劣等行为。“服”出实效，推行“互联网 政务服务”，建成电子政务云平台，开展“减证便民”“数据协同共享”等活动，严格落实绿色通道、并联审批制度，让群众少跑腿、办事更方便。

三、狠抓开放合作，激发经济增长活力

精准促进项目投资。继续深化“项目年”活动，围绕国省政策导向和全市“双六产业”，建立产业链目录和目标企业信息库，抓实项目储备，确保重大项目滚动储备1万亿元以上。加快推进唐家渡电航等138个省、市重点项目建设，完成重点项目投资450亿元以上。打好政策组合拳，统筹好全市招商引资政策和招商项目流转，健全购买招商引资劳务机制，激发委托招商活力，借力商（协）会、企业、合作机构等平台精准招商，加快实施“百企承接行动”，引进投资亿元以上项目100个、到位市外资金500亿元以上。加快推动遂商返乡发展基金投资项目落地，促进广大遂商返乡发展。

主动深化区域合作。积极融入“一带一路”和长江经济带建设。深化成遂协同发展，推动产业园区合作共建，搭好两地企业合作平台，打造成都非省会城市功能疏解拓展区。深化渝遂合作发展，加强与重庆市潼南区等地战略合作，启动遂渝高速扩能改造前期工作，共同推进涪江复航。深化泛成渝经济区商会合作。加强与中科院、四川大学、电子科大等合作，探索发展飞地孵化器，共建产业技术研究院。深化与三亚市、泰国普吉府等国内外友好城市合作，推动互访交流和结对建设。深化对口帮扶理县工作，开创“遂理一家亲”新局面。

大力夯实开放平台。加强外向型园区建设和考核，优化园区开发运营机制，推动西部国际技术合作产业园、欧美产业园、台商工业园、东盟产业园等园区加快建设。优化通关平台，全力争创保税物流中心（B型），加快申建虚拟口岸和国际陆港，支持企业属地报关。加强通道建设，做实与珠海、湛江、宁波等地的“港港合作”，共建西部“无水港”。积极参加2018中外知名企业四川行、西博会、科博会等重大活动，精心举办2018绿色经济遂宁会议、绿色发展科技创新大会、物博会等活动。

四、狠抓名城建设，彰显宜居宜业魅力

内畅外联强基础。联通主动脉，加快推进绵遂内城际铁路、成遂南达万高铁、遂宁动车运用所等重大项目前期工作，开工建设遂德高速等2个高速公路项目、国道350改造等4个干线公路项目，加快遂宁机场迁建等在建项目建设。畅通市内主骨架路网，加快通善大桥升级改造、凤台大桥等项目建设，启动涪江六桥及东西连接线、滨江路至凤台、通港大道建设，实施道路微循环“缓堵保畅”工程，建设城际环线130公里。

建管并重强功能。启动城市总体规划评估与修编，编制“多规合一”专项规划，科学划定城市边界和城市“五线”。加强城市设计，突出“三城”元素，塑造特色风貌。实施城市“双修”，推进原锦华纺织厂、镇江寺片区等区域有机更新。加快市民中心、东湖引水入城等36个项目建设，完成投资40亿元以上。打好海绵城市试点收官战，确保顺利通过国家验收，努力实现由试点变示范。推进“厕所革命”，新建83座、改建216座公厕。宣传贯彻城市管理条例，建立城市管理标准体系，开展城市管理示范创优工程，推进城市管理综合执法力量向街道、乡镇下沉，提高城市管理服务水平。

多城联创强品牌。加强中国观音文化之乡等城市品牌营销，提升遂宁知名度和影响力。统筹推进国家新型城镇化建设试点，建立新型城镇化全程管控体系、绿色智能指标体系，加快20个生态小镇和花园小镇建设。做好全国水生态文明城市建设试点评估验收，全面开展国家生态园林城市创建，争取成功创建国家节水型城市。完成广德、灵泉风景名胜区专项规划编制和中心城区国家湿地公园报批。

五、狠抓乡村振兴，释放三农发展潜力

提升农业。坚持质量兴农、绿色兴农、品牌强农，推进农业供给侧结构性改革十大行动。调优产业体系，落实粮食安全行政首长责任制，提高“3 4 5”产业质效，发展都市型现代农业，确保粮食总产稳定在160万吨、生猪出栏360万头，力争5大市级农业园区引进投资500万元以上的现代农业项目18个，支持船山区创建国家现代农业产业园。调强生产体系，推广农业绿色循环发展新模式，实施“区域品牌 企业品牌”战略，建设“遂宁鲜”营销网点10个，支持射洪县创建国家农产品质量安全县，完成船山区、安居区质量追溯体系建设。调顺经营体系，发展多种形式适度规模经营，推动小农户和现代农业有机衔接，促进农民持续增收。调好服务体系，深化供销合作社综合改革，健全农业社会化服务体系，支持农业机械推广，力争农事服务超市达到16个。

激发活力。激发主体活力，加强基层专业人才队伍建设，开展新型职业农民制度试点，新培育市级以上农业产业化重点龙头企业10家、省级以上农民合作示范社8个、省级家庭农场示范场9个。激发土地活力，加快全面清产核资，支持射洪县抓好国家农村集体产权制度改革试点、蓬溪县开展全省第二批农村集体产权制度改革试点工作，推进承包地“三权分置”改革，力争承包耕地流转面积达到103万亩。激发资金活力，健全适合农业农村特点的农村金融体系，建成市级农村土地经营权信息应用平台，完善农村土地经营权流转履约保证保险机制，深化农村土地承包经营权抵押贷款试点，启动农业产业发展融资风险保障金试点。

建好新村。优化乡村规划，科学制定乡村振兴战略规划，严格落实规划督察员和乡村规划师制度，推广“小规模、组团式、微田园、生态化”，支持有条件的乡村建设田园综合体和特色旅游景区，建成幸福美丽新村200个。优化基础设施，加快推进“农村土坯房三年整治行动”，确保完成整治4.3万户、占总任务的50%。加快蓬船灌区等“6 2”骨干水利工程建设，确保三仙湖水库、祥凤寨水库、萝卜园水库全面完工。加快建设渡改桥、旅游公路以及现代农业园区大环线配套工程，建成县乡道110公里、村道路100公里，新建高标准农田21.85万亩。突出抓好农村垃圾治理、污水处理和厕所改造“三大革命”。优化乡风民俗，传承乡村文脉，保护传统村落，健全自治、法治、德治相结合的乡村治理体系，创建市级及以上“四好村”372个。

六、狠抓生态保护，坚定绿色发展定力

打好污染防治攻坚战。务实整改中央和省级环保督察反馈问题，常态化开展市级环保督察。严格落实减排、抑尘、压煤、治车、控秸等措施，推进臭氧污染防控工作。深化“河长 警长”机制，建立健全乡镇（街道）河湖管理保护机构，加大“一河一策”方案实施力度，全面整治城市黑臭水体，确保郪江、琼江等涪江主要支流水质明显改善。大力实施城镇污水和城乡垃圾处理设施建设三年推进行动，加快射洪县生活垃圾环保发电厂建设。开展集中饮用水水源保护区专项整治，加快渠河饮用水源取水口北移工程建设。严格执行畜禽养殖布局规划，完成限适养区养殖场（户）治理326家。深入开展土壤污染状况详查，开展分类管控和修复。

加大生态系统保护力度。抓好国家生态文明先行示范区建设，加快推进绿色生态屏障等“五大示范工程”。抓好大规模绿化全川遂宁行动，完成人工造林6.3万亩，封育管护2.7万亩，森林覆盖率达到32%。坚持区域环评和项目环评，推动观音湖保护立法，完成涪江（遂宁段）综合保护开发规划编制。强化重点生态功能区管护，制定重点生态功能区产业准入负面清单。开展水土流失综合防治，加快生态环境监测网络建设。

健全生态文明体制机制。制定并实施生态环境损害赔偿制度改革试点方案，加快砂石资源经营管理体制改革和农业水价综合改革，完成环保机构监测监察执法垂直管理制度改革。加强环境保护联合执法、综合执法，健全行政执法与刑事司法衔接机制，严厉打击环境违法行为。严格执行环保述职评议，对行政区域内发生重大及以上突发环境事件实行“一票否决”。

七、狠抓民生实事，提升共建共享能力

打好精准脱贫攻坚战。扎实开展“回头看、回头帮”。今年要再实现90个贫困村退出、2.5万贫困人口脱贫。制定并实施交通、水利、旅游等24个扶贫专项年度实施方案，健全脱贫攻坚常态长效机制。持续抓好产业增收，推进产业扶贫大环线建设和产业融合发展，带动贫困户人均增收1000元以上。突出抓好住房安全，完成省下达2888人易地扶贫搬迁任务。大力抓好教育扶智、就业扶能、健康扶贫，努力实现“人人有学上、个个有技能、家家有希望”。扎实抓好基础扶贫，推进“四好农村路”建设，统筹解决生产生活用水、安全用电、广播电视、通信网络等问题。保持“四项基金”合理规模，更好发挥基金的特殊作用。深化农村集体产权制度改革和资产收益扶贫试点，稳定增加村集体资产性收入和经营性收入。鼓励支持社会力量参与扶贫，形成专项扶贫、行业扶贫、社会扶贫“三位一体”大扶贫格局。注重扶贫同扶志、扶智相结合，开展好扶贫领域“作风建设年”活动，深化扶贫领域监督执纪问责工作，确保脱贫成果得到群众认可、经得起历史检验。

提高保障和改善民生水平。办好民生工程和民生实事。实施积极的就业创业政策，城镇新增就业3.3万人，城镇登记失业率控制在4.4%以内。持续推进“百姓安居工程”，改造城市棚户区18894套。实施城乡教育振兴等“六大工程”，提高公办教育质量，加快科教园区建设，启动遂宁技师学院筹建，深化职业教育现代学徒制试点，推进特殊教育融合发展，让每个孩子“幼有所育、学有所教”。抓好大健康产业发展，积极发展以本草文化为特色的中医药产业。完善分级诊疗制度，建设新型城乡医联体，扩大免费健康体检试点，深化医保支付方式改革，做实家庭医生签约服务，加快市中心医院河东院区二期工程等项目建设，让群众就医更方便、更实惠。巩固全民参保登记成果，妥善做好被征地农民社会保障工作。提高城乡低保、特困供养标准，稳步发展养老服务产业，扎实开展居家和社区养老服务改革试点。大力发展慈善事业，抓好殡葬基础设施建设和惠民殡葬服务，深化残疾人“量体裁衣”式服务。积极备战省运会，推进体育场馆管理体制改革，构筑全民健身网络体系，让市民有地可锻炼、随处可健身。

繁荣发展文化事业。加快宋瓷文化中心建设，完善四级公共文化服务体系。实施巴蜀文化品牌工程、文艺精品创作工程，创作出版《遂宁史画》，办好遂宁国际诗歌周暨陈子昂诗歌奖、观音文化旅游节、涪江文化艺术节等活动。加强文化遗产保护传承和利用，支持大英卓筒井申报世界文化遗产，发展民办博物馆，鼓励遂宁杂技、曲艺、宋瓷、观音工艺品等特色文化产品开展对外交流。健全现代文化产业体系和市场体系，加快“六大文化产业园区”建设，推动印纪传媒、博宇传媒、天遂文旅等企业做大做强。拓展“高清遂宁智慧广电”，加强文化市场监管，倡导全民阅读，建设文化强市。

八、狠抓社会治理，凝聚共治共管合力

防范化解重大风险。完善金融联席会议制度，加强隐患摸排和风险防控。建立政府、银行、企业联动机制，深化债委会工作，增强企业债务风险化解实效。加强民间融资风险防控和处置，持续深入开展“打非治赖”专项行动，依法防控、打击和处置非法集资。逐步建立多主体供给、多渠道保障、租购并举的住房制度，保持房地产市场平稳健康发展。规范政府举债行为，落实政府性债务全口径监管机制，严格债务限额管理，坚决反对劳民伤财、急功近利的政绩工程。

提高社会治理水平。巩固“长安杯”创建成果，提高社会治理社会化、法治化、智能化、专业化水平，打造共建共治共享格局。强化“三社”联动，调动居民自治积极性，培育壮大社会组织，开展专业化社会服务，支持工会、共青团、妇联、关心下一代等群团组织深化改革和参与社会治理。深化司法体制综合配套改革，加大“法律七进”和基层法治示范创建力度。完善“诉非衔接”机制，继续执行领导包案制度，深入化解八大类民生突出问题。健全“治欠保支”工作机制，构建和谐劳动关系。加强信用体系建设，健全守信联合激励和失信联合惩戒制度。

加强公共安全管理。强化社会治安防控和公共安全体系建设，扎实开展扫黑除恶专项斗争，保持对各类违法犯罪的严打高压态势。严格落实安全生产责任制，坚决遏制重特大事故发生，力争新创建省级安全社区4个。强化食品药品安全监管，建成标准化基层食品药品监管所40个，支持射洪县创建省级食品安全示范县。完善防灾减灾和应急管理体系，建成遂宁市突发公共事件预警信息发布系统，提升应急救助能力。加强国防动员、优抚安置、双拥、对台、侨务和外事等工作。

加强政府自身建设

打铁必须自身硬。全市各级政府将深入学习贯彻党的十九大精神，以习近平新时代中国特色社会主义思想统领各方面工作，把人民对美好生活的向往作为奋斗目标，从严治政、依法行政、廉洁从政，推动全市政府系统干部更加自觉地为广大人民群众谋利益。

始终保持忠诚之心。忠诚于组织，扎实开展“不忘初心、牢记使命”主题教育，牢固树立“四个意识”，坚决维护以习近平同志为核心的党中央权威和集中统一领导。忠诚于人民，深入一线察民情、解民忧，认真办好“12345”政府服务热线，持续整治“群众最不满意的10件事”，创新整治“企业最不满意的10件事”。忠诚于职守，把确保上级和市委政令畅通、令行禁止作为重大政治责任，不折不扣落实省委、省政府及市委决策部署。

始终保持公道之心。严格依法办事，自觉运用法治思维和法治方式推进政府工作，加强政府决策合法性审查，全面推行执法全过程记录、重大执法决定法制审核和行政执法公示制度。坚持诚信干事，推行阳光政务，加强政府采购、招商引资等六大领域政务诚信建设，保持重要规划、重大政策和重点工作的连续性，依法履行对群众、企业和投资者的各项承诺，构建“亲”“清”新型政商关系。推动和谐共事，严格执行民主集中制，坚持“三重一大”决策制度，集思广议抓好工作落实。

始终保持进取之心。积极主动作为，大兴调查研究之风，强化执行力建设，健全落实重大决策部署的督查考核办法，努力激发干部干事创业激情。善于创新作为，探索创新新时代富民强市工作方法和经验，在“七大提升行动”“三大攻坚战”等领域形成一批创新亮点。努力匠心作为，大力弘扬专业精神，建立健全提升专业素养和学历层次的激励机制，推动干部队伍全面加强学习和实践，培养一批“行家里手”。

始终保持敬畏之心。广泛接受监督，自觉接受人大及其常委会法律监督和工作监督，自觉接受政协民主监督，认真办理人大代表建议和政协委员提案，主动听取民主党派和社会各界的意见，主动接受社会和舆论监督，推进审计监督全覆盖，支持监察监督和司法监督。深化作风建设，深入贯彻中央八项规定及实施细则精神，全面落实省、市十项规定及实施细则要求，扎实开展“作风建设年”活动，坚决纠正“四风”，持续推进精文简会高效办事，强力整治机关效能和发展软环境问题，倾力打造一流政务环境。强化制度约束，健全重点领域、重大项目、关键环节、重要岗位运行机制，严肃查处群众身边的不正之风和腐败问题，扎紧廉洁从政的制度“笼子”。

各位代表，新时代开启新征程，新使命呼唤新作为。让我们更加紧密地团结在以习近平同志为核心的党中央周围，在省委、省政府和市委的坚强领导下，锐意进取，埋头苦干，为决战决胜全面小康、建设绿色经济强市、实现美丽繁荣和谐遂宁目标作出新的更大贡献！
